# Disturbances of Ruminal Microbiota and Liver Inflammation, Mediated by LPS and Histamine, in Dairy Cows Fed a High-Concentrate Diet

**DOI:** 10.3390/ani14101495

**Published:** 2024-05-17

**Authors:** Nana Ma, Junfei Guo, Zhenfu Li, Lei Xu, Kai Zhang, Tianle Xu, Guangjun Chang, Juan J. Loor, Xiangzhen Shen

**Affiliations:** 1 Ministry of Education Joint International Research Laboratory of Animal Health and Food Safety, College of Veterinary Medicine, Nanjing Agricultural University, Nanjing 210095, China; t2022058@njau.edu.cn (N.M.); junfeiguo91@gmail.com (J.G.); 13270733639@163.com (Z.L.); njauxulei@163.com (L.X.); zhangkai11895452@126.com (K.Z.); tl-xu@outlook.com (T.X.); gjchang@njau.edu.cn (G.C.); 2 Department of Animal Sciences, Division of Nutritional Sciences, University of Illinois, Urbana, IL 61801, USA; jloor@illinois.edu

**Keywords:** high-concentrate diet, ruminal microbiota, LPS, histamine, liver

## Abstract

**Simple Summary:**

Ruminal microbiota plays an important role in the health and milk production of dairy cows. In this study, we found that high-concentrate diet (concentrate: forage = 60:40) feeding altered the composition of the microbiota in the rumen and produced toxic metabolites (LPS and histamine). The elevated concentrations of LPS and histamine in the gut were transferred into the bloodstream and caused damage to the ruminal and cecal walls. LPS and histamine stimulated an inflammatory response in the liver with the increased presence of H1 receptors. Our study provides a basis for exploring the impact of changes in gut microbiota composition on animal health and offers a novel therapeutic strategy for treating metabolic disorders caused by SARA.

**Abstract:**

The ecosystem of ruminal microbiota profoundly affects the health and milk production of dairy cows. High-concentrate diets are widely used in dairy farms and evoke a series of metabolic disorders. Several studies have reported the effects of high-concentrate diets on the ruminal microbiome, while the effect of changes in ruminal microbial flora, induced by high-concentrate diet feeding, on the liver of dairy cows has not been studied before. In this study, 12 mid-lactating Holstein Friesian cows (weight of 455 ± 28 kg; parities of 2.5 ± 0.5; starting milk yield of 31.59 ± 3.2 kg/d; DMI of 21.7 ± 1.1 kg/d; and a DIM at the start of the experiment of 135 ± 28 d) were fitted with ruminal fistulas, as well as with portal and hepatic vein catheters. All cows were randomly divided into 2 groups; then, they fed with low-concentrate diets (LC, concentrate: forage = 40:60) and high-concentrate diets (HC, concentrate: forage = 60:40) for 18 weeks. The forage sources were corn silage and alfalfa hay. After the cows of two groups were euthanized over two consecutive days, ruminal microbiota; the concentration of LPS in the rumen content; cecum content; the levels of blood and histamine in rumen fluid, blood, and the liver; the histopathological status of the rumen and cecum; and the inflammatory response of the liver were assessed in dairy cows under conditions of subacute ruminal acidosis (SARA). These conditions were caused by high-concentrate diet feeding. All data were analyzed using the independent *t*-test in SPSS. The results showed that high-concentrate diet feeding increased the concentration of LPS and histamine in the rumen and plasma of veins (*p* < 0.05). The abundance of *Bacteroidetes* at the phylum level, and of both *Bacteroidetes* and *Saccharibacteria* at the genus level, was decreased, while the abundance of *Firmicutes* at the phylum level and *Oscillibacter* at the genus level was increased by high-concentrate diet feeding. The decreased pH values of ruminal contents (LC = 6.02, HC = 5.90, *p* < 0.05) and the increased level of LPS in the rumen (LC = 4.921 × 10^5^, HC = 7.855 × 10^5^ EU/mL, *p* < 0.05) and cecum (LC = 11.960 × 10^5^, HC = 13.115 × 10^5^ EU/mL, *p* < 0.01) induced the histopathological destruction of the rumen and cecum, combined with the increased mRNA expression of IL-1β (*p* < 0.05). The histamine receptor H1R and the NF-κB signaling pathway were activated in the liver samples taken from the HC group. In conclusion, the elevated concentrations of LPS and histamine in the gut may be related to changes in the ruminal microbiota. LPS and histamine induced the inflammatory response in the ruminal epithelium, cecum epithelium, and liver. However, the cause–effect mechanism needs to be proved in future research. Our study offers a novel therapeutic strategy by manipulating ruminal microbiota and metabolism to decrease LPS and histamine release and to improve the health of dairy cows.

## 1. Introduction

The gut microbiome consists of bacteria, archaea, fungi, and protozoa. It is usually studied in human diseases, such as gastroenteritis and colitis. However, in recent decades, microbiota-related research has expanded and focused on host physiology and health [1]. Ruminants rely on their gut microorganisms to digest the complex polysaccharides they receive from the plants, such as cellulose and hemicellulose, into microbial biomasses and nutritive foods, such as acetate, propionate, and butyrate [2]. The development of the rumen epithelium is strongly affected by the function of the ruminal microbiome [3]. The ruminal microbiome and ruminal metabolome contribute partially to the milk yield of a host [4]. Diet, age, gender, genetics, and geographical location determine the composition and function of the ruminal microbiome [5]. High-feed-efficiency cows display lower diversity and richness of ruminal microbiome, indicating that reducing the proportion of non-essential microorganisms can improve feed efficiency [6]. The ruminal microbial community is also associated with the cow’s performance and milk production during the peripartal period [7]. Therefore, the effect of the ruminal microbiome on the health and physiology of the host needs to be explored further.

A high-concentrate diet is usually selected for high milk production in intensive dairy farms. A high-concentrate diet can induce subacute ruminal acidosis (SARA), which often occurs along with low ruminal pH values. SARA induces a series of metabolic diseases, such as the inflammatory response in the mammary gland, milk fat depression [8], and laminitis [9]. Our previous studies also showed that high-concentrate diet feeding induced hepatocyte lesions, immune defense responses [10], and oxidative stress [11]. The increased proportion of starch-degrading bacteria and the decreased proportion of fiber-degrading bacteria might be the reason for the susceptibility to SARA [12]. This is a common clinical problem that needs to be solved urgently [13], and SARA is a typical case model of a ruminal microflora disorder in cows. SARA reduces the richness and diversity of the microbiome in ruminal fluid, but has no effect on cecal digesta [14]. A grain-based SARA challenge only alters bacterial communities in ruminal fluid with lower taxonomical levels, except for in *Firmicutes* [15]. Thus, in this study, we try to explore the change in the composition of ruminal microbiomes during SARA, which is caused by long-term high-concentrate diet feeding.

When dairy cows are fed with high levels of forage, the diversity of the microbiome in the rumen is similar to that found in the cecum and colon, while this changes [16] when the animal is fed with a high-concentrate diet. Ruminal microbiota is related to the occurrence of laminitis [17] and mastitis [18] in dairy cows. SARA also affects animal metabolite levels through byproducts such as LPS. This is mainly produced by *Proteobacteria*, *Bacteroidetes*, and *Fibrobacteres* when they are in a logarithmic phase, and under conditions of cell disintegration and lysis [19,20]. The production of LPS and histamine by ruminal flora increases the risk of developing metabolic diseases [21]. Most studies only focus on the effect of high-concentrate diets on ruminal microbiota.

Thus, we hypothesized that the microbiota disorder caused by SARA induces the inflammatory response of the liver and we provided evidence for the influence of gut microbiota on the health of dairy cows. In this study, SARA was induced in dairy cows fed on a high-concentrate diet for 18 weeks. The ruminal microbiota was analyzed using a 16s RNA sequence and the concentration of toxic metabolites, LPS, and histamine, the presence of which was detected in ruminal content and bloodstream. The histopathological changes in the rumen and cecum and the inflammatory damage in the liver were evaluated.

## 2. Materials and Methods

### 2.1. Ethics Statement

The animal experiments were examined and approved by the Animal Care and Use Committee of Nanjing Agricultural University, and all animal operations strictly complied with the Animal Experiment Guidelines issued by the Ministry of Science and Technology (Beijing, China).

### 2.2. Animals and Experimental Design

Twelve multiparous lactating Holstein Friesian cows (weigh of 455 ± 28 kg; starting milk yield of 31.59 ± 3.2 kg/d; DMI of 21.7 ± 1.1 kg/d; DIM at the start of the experiment of 135 ± 28 d; 2–3 parities) were selected. In this experiment, we installed them with ruminal fistula, portal, and hepatic vein catheters [22,23]. After general anesthesia, the cow was placed on its left side. A 30 to 40 cm paracostal incision was made on the right side of the abdomen. It was located 4 to 6 cm from the lumbar spine, was perpendicular to the spine, and was caudal to the last rib, running through skin and muscles. For hepatic vein catheterization, the liver should be touched gently to locate the hepatic vein. The main branch of the hepatic vein was found in the middle of the right lobe of the liver. We inserted the 20# steel needle into the hepatic vein. When the hepatic venous blood was drained, the silicone rubber catheter (1.5 mm*3.2 mm, Chensheng Medical, Jinan, China) was inserted through the needle, making sure the tip of the catheter was in the hepatic vein, not in the abdominal vein. After the needle was removed, three stitches with silk were made to fix the catheter onto the diaphragmatic surface of the liver. A tubing adapter was inserted into the catheter, which was filled with heparinized saline and checked for patency. For the portal vein catheterization, the portal vein collected blood from the stomach, the small intestine up to the ileum, the hind-gut, the spleen, and the pancreas. The branches of the portal vein were deeper than those of the hepatic vein. The portal vein branch was located between the hepatic vein and the gallbladder, and the steel needle was inserted parallel to the hepatic vein branch into the portal vein branch through the liver tissue until blood gushed out. The surgeon placed a finger under the portal vein in advance and inserted the catheter through the steel needle into the portal vein until the tip of the catheter reached the finger to make sure that it was in the portal vein. The catheter was then fixed with three stitches. The patency of the catheter filled with heparinized saline was checked regularly during catheterization. At the end of two surgeries, the catheters were threaded away through the muscle and skin and secured on the backs of the cows. All cows had a recovery period of two weeks.

For a rumen fistula, an incision of 15–20 cm in length was made at the midpoint of the last rib and hip nodule, and we cut under 5–8 cm the lumbar shelf on the left side of cows. Rumen fistulas (7.5 cm, Anscitech, Wuhan, China) were installed on the dorsal side sac of the rumen, and the rumen serosal muscle layer was sutured with the internal and external purse strings, and finally the muscle and skin were sutured. Antibiotics were injected intramuscularly every day for 7 days, and the wounds and fistulas were wiped with iodine tincture daily. After fasting for more than 36–48 h, a small amount of forage was fed after rumen peristalsis and rumination was recovered. When they had fully recovered from surgery after about 2 weeks, cows were fed for 4 weeks with a low-concentrate (LC) diet that was adapted to the environment. This diet consisted of 60% forage and 40% concentrate.

Then, cows were randomly divided into two groups. Six cows were fed with a high-concentrate (HC) diet, which contained 40% forages and 60% concentrate, as the HC group. The other six cows remained on the LC diet as the LC group. Sample size determination was based on the assumed LPS concentration of the portal vein plasma (the LC group is 0.1 EU/mL and the HC group is 0.2 EU/mL, *p* < 0.05, 95% power), as determined using an online tool (https://www.bu.edu/researchsupport/compliance/animal-care/working-with-animals/research/sample-size-calculations-iacuc/, accessed on 30 April 2024) [8]. All cows were kept in individual tie stalls and had free access to fresh water throughout the experiment. Cows were fed at 04:00, 12:00, and 20:00 for 18 weeks based on our previous study, with decreased milk protein (from week 15), increased SCC (from week 9) [24], and decreased milk yield (from week 7) [11]. The pH of the ruminal liquid was recorded every week. The ingredients and nutritional composition of the diets are presented in Table 1. The two diets had similar levels of NEI and CP. Corn silage and alfalfa hay formed the forage portion, and the other ingredients constituted the concentrate portion.

### 2.3. Sample Collection

Ruminal fluid was extracted through the ruminal fistula before morning feeding (0 h, at 04:00) and after feeding, with 1 h intervals left before the second feeding (12:00) on the last day of the eighteenth week. The collected samples were filtered through 2 layers of gauze. Then, their pH values were tested directly using a pH meter (HI 9125; Hanna Instruments, Smithfield, RI, USA) and the 4 h samples were stored at −20 °C for LPS and histamine measurement later on. Blood samples were collected 4 h after feeding on the last day of the eighteenth week. The blood was taken via portal and hepatic vein catheters and from the jugular vein into 5 mL vacuum tubes containing sodium heparin. Plasma was isolated via centrifugation at 3000× *g* at 4 °C for 15 min and stored at −20 °C for the measurement of LPS, histamines, or inflammatory cytokines. At the same time, the ruminal content was taken through the ruminal fistula and used for 16s RNA sequence.

After the experimental period, cows from the LC group and the HC group were euthanized using a captive bolt after anesthetization on two consecutive days. We took samples of rumen epithelium from the ventral sac and cecum epithelium. These were rinsed with saline and subsequently stored at −80 °C for subsequent laboratory testing. The cecum content was collected and stored at −20 °C for histamine determination. Whole-thickness rumen samples from the ventral sac and cecum tissues (approximately 1 cm^2^) were fixed in a 4% paraformaldehyde solution for histological analysis. The liver was taken and cut into small pieces and then rinsed with saline for storage at −80 °C for use in subsequent laboratory testing. All sample collecting operations were finished within 30 min of the captive bolt.

### 2.4. LPS Measurement

The LPS concentrations in the ruminal liquid, cecum content, plasma of the portal vein, and plasma of the hepatic vein were measured. The sample preparation of plasma for LPS measurement was reported previously [8]. The cecal contents (1 g) were diluted in 1 mL of 0.9% saline in a pyrogen-free tube and thoroughly mixed to obtain cecum liquid. The liquids of the rumen and cecum were centrifuged at 10,000× *g* for 45 min, and then the supernatant was passed through a disposable 0.22 μm pyrogen-free filter. The filtrate was boiled at 100 °C for 30 min. After being diluted to a suitable concentration, the LPS concentration of all samples was tested using the Chromogenic End-point Tachypleus Amebocyte Lysate Assay Kit (Chinese Horseshoe Crab Reagent Manufactory Co., Ltd., Xiamen, China) according to the manufacturer’s instructions. The kit had a sensitivity of 0.01 EU/mL. The standard curve of the LPS concentration (y) and OD value (x, at 545 nm) was y = 4.7586x − 0.0089, with R^2^ = 0.9927. The LPS concentration in the samples was calculated using the standard curve.

### 2.5. Histamine Measurement

The collected liver tissue was kept in liquid nitrogen and ground to a powder using a mortar. The liver sample was added to a 1× PBS (100 mg into 1 mL) solution, homogenized using a homogenizer, and centrifuged at 3000× *g* for 10 min; then, the supernatant was collected. Ruminal liquid and plasma from the jugular vein were added into a 1× PBS (100 μL into 200 μL) solution. The histamine concentration of all samples was detected using a double-antibody sandwich enzyme-linked immunosorbent assay kit (H171, Jiancheng Bioengineering Institute, Nanjing, China) according to the instructions of the manufacturer. We added samples and standard histamine solution (concentration from 1600 ng/mL to 100 ng/mL) into wells that were pre-coated with bovine HIS monoclonal antibody. After incubation, histamine antibodies labeled with biotin and Streptavidin labeled with HRP were added into the well separately. After the addition of chromogen solutions A and B, the OD value of each well at 450 nm was measured using a full-wavelength microplate photometer (Thermo Fisher Scientific Inc., Waltham, MA, USA). The histamine concentration in the samples was calculated using the standard curve.

### 2.6. Histological Analysis

Rumen and cecum tissues, which were fixed with 4% paraformaldehyde, and then subjected to dehydration, transparency, wax immersion, embedding, slicing, slide making, and other processes, were finally fixed on the slide. Hematoxylin-eosin staining (HE staining) was observed under a microscope after the staining was added with gum and sealed with a coverslip. Ruminal papillae and the wall were examined separately.

### 2.7. Radioimmunoassay

The concentrations of inflammatory cytokine IL-1β, IL-6 and TNF-α in the plasma of the hepatic vein was measured with radioimmunoassay kits (IL-1β, C09DJB; IL-6, C12DJB; TNF-α, C06PJB; Beijing North Institute of Biological Technology, Beijing, China) using a gamma radioimmunoassay counter (Shanghai Hesuo Rihuan Photoelectric Instrument Co., Ltd., Shanghai, China).

### 2.8. Quantitative Real-Time PCR

The total RNA of rumen epithelium, cecum epithelium, and liver were extracted with Trizol (9108, Takara, Otsu, Japan) according to the manufacturer’s protocol. The quantity and quality of RNA were determined using a NanoDrop ND-1000 Spectrophotometer (Thermo Scientific, Waltham, MA, USA), and RNA integrity was detected using 1% agarose gel electrophoresis [11,25]. Hifair III 1st Strand cDNA Synthesis Super Mix for qPCR (11141ES60, Yeasen, Shanghai, China) was used for the reverse transcription of total RNA to cDNA, and cDNA was diluted four times. A 10 μL system was prepared using the ChamQ Universal SYBR qPCR Master Mix (Q711, Vazyme, Nanjing, China) according to instructions, and the RT-qPCR program was run by ABI 7300 Fast Real-Time PCR system (Applied Biosystems, Foster City, CA, USA). The mRNA sequence of the target gene was found on the NCBI website, and primers were designed using Premier 6.0 (Premier Bio-Soft International, San Francisco, CA, USA). The primer list was the same as in the previous studies [8,26]. In this study, GAPDH was used as the reference gene to correct the expression of target genes. The relative expression levels of target genes were calculated using the 2^−ΔΔCt^ method. The final results were presented as the fold change relative to the mean value of the control.

### 2.9. Western Blot Analysis

Approximately 100 mg of the ground liver sample was weighed and added into 1 mL of RIPA working solution (RIPA: PMSF = 1:100) in an ice bath. After homogenization with a Dounce homogenizer, samples were kept on ice for 10 min and then centrifuged at 12,000× *g* for 20 min at 4 °C to obtain the supernatant. After denaturing with 5× SDS loading buffer (EpiZyme, Shanghai, China) in 99 °C water bath, the target proteins were separated in a 10% SDS-PAGE gel. Then, the blots of the target protein were transferred onto the PVDF membrane. The membrane was bolted in 7% skim milk, and incubated in primary antibodies (NF-кBp65, Cat. AN365, 1:1000, Shanghai, China; NF-кBpp65, Cat. AN371, 1:1000, Beyotime Biotechnology Co., Ltd. Shanghai, China; β-actin, Cat. SC130656, Santa Cruz Biotechnology, Dallas, TX, USA, 1:500) and HRP-conjugated secondary antibodies separately. The blot of target protein was visualized with an ECL kit, and the signal was captured by ChemiDoc TM XRS + Image System (Bio-rad, Berkeley, CA, USA). Image Lab Software (Bio-rad, Berkeley, CA, USA) was used to analyze the intensity of each band. The final result of each target protein was presented as relative abundance to reference the protein β-actin.

### 2.10. DNA Extraction, 16S rRNA Gene Amplification, Ion S5TM XL Sequencing, and Data Analysis

After the ruminal contents were pulverized with a 2010 Geno/grinder, the microbial DNA was extracted from the ruminal contents using a PowerSoil-htp 96 Well Soil DNA Isolation Kit (Cat. 12955, MO BIO Laboratories, Inc., Carlsbad, CA, USA) according to the kit’s instructions. The purity and concentration of DNA samples were checked using a NanoDrop ND-1000 Spectrophotometer (Thermo Scientific, Waltham, MA, USA) and agarose gel electrophoresis, and the DNA samples were diluted with ddH_2_O to a uniform concentration of 0.5 ng/μL.

Ruminal microbial communities were analyzed by sequencing the region of the bacterial 16S rRNA gene. Universal primers with 6 bp barcodes were used to amplify the V3 and V4 regions of the 16S rRNA gene of each sample. The primer sequences were 341F (5′-CCTACGGGNGGCWGCAG-3′) and 785R (5′-GACTACHVGGGTATCTAATCC-3′). PCR amplification was run using the GoTaq^®^ Hot Start Colorless Master Mix (M5133, Promega, Madison, WI, USA) apparatus and the procedure was as follows: 95 °C 2 min (first pre-degeneration), 95 °C 30 s repeated for 25 cycles (denaturation), 55 °C 30 s (Annealing), 72 °C 30 s (elongation), and 72 °C 5 min (extension). According to the concentrations of PCR products, the samples were mixed in equal concentrations, and detected using 2% agarose gel electrophoresis after thorough mixing. The mixed PCR products were purified and recovered using a QIAquick^®^ PCR Purification Kit (28106, Qiagen, Hilden, Germany).

All amplicon libraries were sequenced using an Illumina MiSeq platform (San Diego, CA, USA), and the raw sequences were processed using QIIME. The assembled reads were demultiplexed according to the barcode sequences, and chimeric reads were filtered. Sequence quality control was performed as follows: the sequences with an average mass fraction of 10–20 bp were cut at any base point of the 250 bp sequence, while the sequences with a length of less than 50 bp and the sequences containing ambiguous bases and mismatched bases were discarded. Clean reads were clustered into OTUs (operational taxonomic units) based on 97% similarity with UCLUST. Representative sequences from each OTU were assigned a taxonomy using Ribosomal Database Project (RDP) Classifier 2.3. OTUs were divided by the Mothur v.1.29.0 software, and OUT abundance spectra were generated according to the number of sequences in question. The relative abundance of the two groups was analyzed at the phylum and genus levels, respectively. The top 5 phyla and the top 30 genera were selected in order to generate the bar chart. The alpha diversity values, including Chao1, ACE, Shannon, and Simpson, were used to evaluate the complexity of species richness and the diversity of each sample. The alpha rarefaction curves were constructed to that ensure sufficient sequencing depth was achieved. We used principal coordinate analysis (PCoA) of unweighted UniFrac analysis as the beta diversity in order to evaluate the distance between the ruminal contents in LC and HC groups. LEfSe was used to identify bacterial taxa that are differentially represented between ruminal microbiota from two groups, and linear discriminant analysis (LDA) was used to estimate the magnitude of the effect of the abundance of each bacterial taxa on the differences between the two groups.

### 2.11. Statistical Analysis

The residuals for each variable were used to assess normality. The statistical differences between groups were analyzed using the independent *t*-test in SPSS 20.0 (IBM Corp., Armonk, NY, USA). In this study, *p* < 0.05 was considered a significant difference. All data are expressed as the means ± SEM. Figures were drawn using GraphPad Prism 6.01 (GraphPad Software, Inc., San Diego, CA, USA).

## 3. Results

### 3.1. SARA Is Induced by a High-Concentrate Diet Combined with High Production of LPS and Histamine in Gut and Bloodstream

The primary characteristic of SARA is the decreased pH level in the rumen. The daily average pH in the HC group was significantly lower than that in the LC group, and the duration time of pH < 5.6 in the HC group was 223 min more than 3 h (Table 2). Thus, the SARA model was successfully established in a manner consistent with the previous study [27]. The concentration of LPS in the rumen and cecum contents increased markedly in the HC group compared with the LC group. Meanwhile, the concentration of LPS in plasma of the portal vein also clearly increased in the HC group, while no significant difference was found in the plasma of the hepatic vein between the two groups (Table 2). Thus, we speculated that high-concentrate diet feeding increases the production of LPS in rumen, which is transferred through the ruminal barrier to the liver via the hepatic vein, and the liver clears the increased LPS in HC group. Histamine is the other important toxic metabolite produced by ruminal microbiota. The concentration of histamine in the ruminal liquid and jugular vein of HC group was significantly higher than that in the LC group. The level of histamine in the liver tissue was also increased markedly in the HC group (Table 3). The results showed that histamine also accumulated in the rumen and transferred through the ruminal barrier to the liver.

### 3.2. The Microbiota Composition Is Changed in the Rumen under SARA

**Figure 1:**
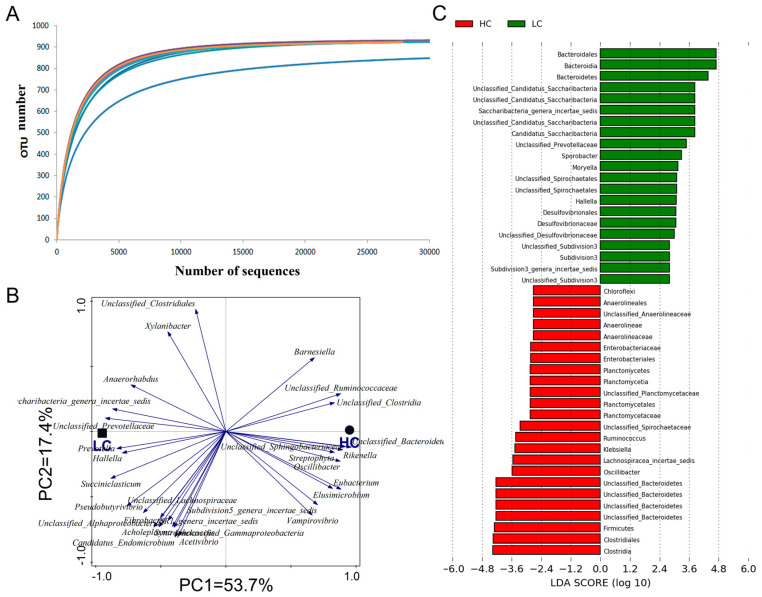
The richness, diversity, and LEfSe analysis of microbiota taken from ruminal contents between two groups. (**A**) Rarefaction curve. (**B**) PCoA analysis of microbiota at the genus level. The abscissa represents the first principal component, and the percentage indicates the contribution of the first principal component to sample difference. The vertical axis represents the second principal component, and the percentage indicates the contribution of the second principal component to sample difference. Solid square, low-concentrate diet. Solid circle, high-concentrate diet. Arrows refer to species that have a higher correlation with principal component values, while length of arrows indicate the correlation values. The species that arrow points to indicates a higher relative abundance of the species. (**C**) Linear discriminant analysis (LDA) score derived from LEfSe analysis. The length of the histogram represents the contribution of taxa on difference between two groups. Green color, low-concentrate diet. Red color, high-concentrate diet.

Rarefaction curves of 12 samples tend to flatten out, indicating that the sampling depth was sufficient to depict the bacterial abundance (Figure 1A). Alpha diversity was used to compare the bacterial abundance and diversity between the LC and HC group. There was no difference in the estimators of community richness (Chao 1 and ACE) and diversity (Shannon index and Simpson index) between the two groups (Table 4). Bacteria with higher relative abundance in the HC group were mainly located on the right side of the first principal component, PC1, while the bacteria with higher relative abundance in the LC group were mainly located on the left side of PC1. This indicated that the high-concentrate diet had a significant effect on the microbiome of ruminal contents (*p* = 0.014) (Figure 1B).

**Figure 2:**
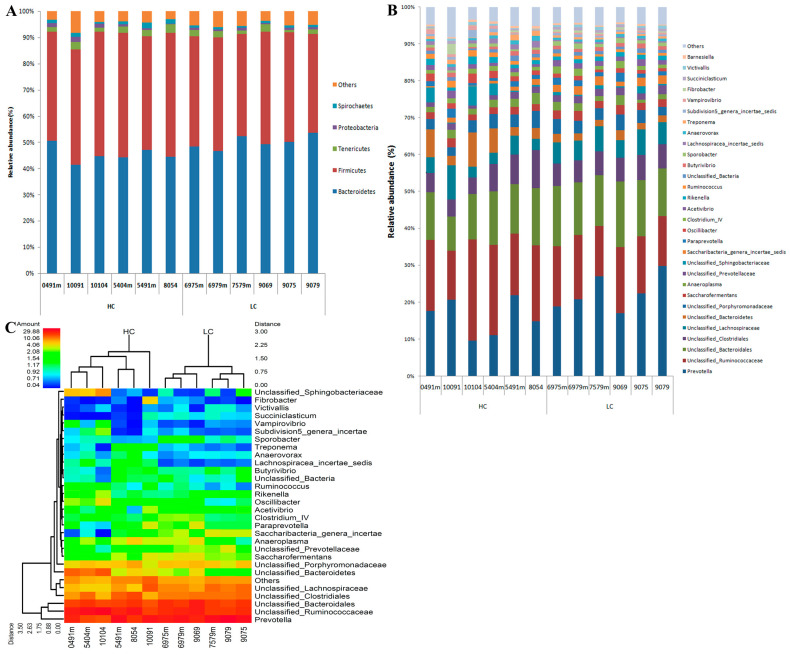
The relative abundance of dominant Phylum and Genus of microbiota in ruminal contents. (**A**) showed the highest relative abundance of 5 microbiota at phylum level and (**B**) showed the highest relative abundance of 30 microbiota at genus level in ruminal contents of two groups. The abscissa distribution shows each sample, the ordinate shows the percentage of the relative abundance of each strain. Different colors represent different strains. (**C**) the heat map of top 30 microbiota at genus level. The horizontal information represents the samples, and the species annotation information is vertical. On the same branch of the sample cluster tree on the left indicating that the species distribution is similar. The colors of the figure correspond to the relative abundance of each species. Red stands for a large number of genera, while blue represents fewer. HC, high-concentrate group. LC, low-concentrate group.

The relative abundance of the two groups was analyzed, respectively. The top five phyla and the top 30 genera were chosen in order to create a bar chart (Figure 2A,B). At the phylum level, *Bacteroidetes* (46.58%) and *Firmicutes* (43.58%) were the dominant bacteria in the two groups, followed by *Tenericutes*, *Proteobacteria*, and *Spirochaetes.* The abundance of *Bacteroidetes* at the phylum level in the HC group was significantly lower than that in the LC group. Among these groups, the number of *Hallella* (*p* < 0.01), *Prevotella* (*p* = 0.04), and unclassified *Prevotella* (*p* = 0.01) in the *Prevotella* family were significantly lower, but the number of unclassified *Bacteroidetes* was significantly higher (*p* = 0.027). Compared with the LC group, the content of *Firmicutes* in the HC group increased significantly, including unclassified *Lachnospiraceae* (*p* < 0.001), *Oscillibacter* (*p* = 0.007), *Ruminococcus* (*p* = 0.015), and unclassified *Clostridia* (*p* = 0.037). However, there were significant decreases in the rates of *Sporobacter* (*p* = 0.012) and *Succiniclasticum* (*p* = 0.006). In addition, *Planctomycetaceae* (*p* = 0.022) and *Spirochaetaceae* (*p* < 0.001) were significantly higher in the high-concentrate group than in the low-concentrate group (Table 5). The heat map was made for the top 30 genera with the highest relative abundance in the ruminal contents (Figure 2C). The genera *Prevotella*, the unclassified *Ruminococcaceae*, and the unclassified *Bacteroidales* clustered into one cluster, and the remaining 27 genera clustered into the other cluster. The relative abundance of *Saccharibacteria*, unclassified *Prevotellaceae*, *Butyrivibrio,* and *Succiniclasticum,* in the HC group decreased, while that of unclassified *Bacteroidetes*, *Oscillibacter*, *Rikenella*, *Ruminococcus,* and *Sphingobacteriaceae* increased in the HC group compared with the LC group. Microbial communities with significant differences in tems of abundance were detected and identified using LEfSe, and linear discriminant analysis (LDA) was used to estimate the effect of each species on the difference between the two groups. *Bacteroidetes* and *Saccharibacteria* were the main bacterial groups, indicating that the relative abundance of *Bacteroidetes* was higher in the low-concentrate group than in the high-concentrate group. *Clostridia* and *Ruminococcus* were the main bacteria that caused the relative abundance of *Firmicutes* in the HC group to be significantly higher than that in the LC group (Figure 1C).

### 3.3. SARA Induces Histopathological Changes and Inflammatory Responses in the Rumen and Cecum

**Figure 3:**
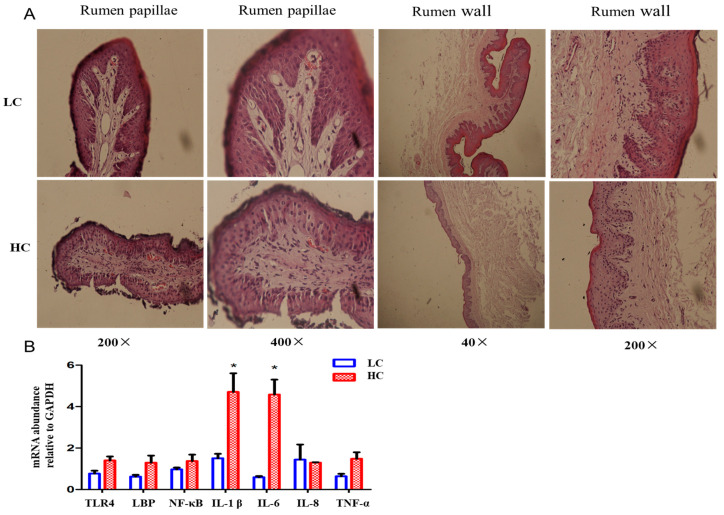
High-concentrate diet caused histopathological changes and inflammatory responses in the ruminal papilla and ruminal wall. (**A**) Representative sections of the ruminal papillae and wall from two groups. All sections were stained with H&E and observed at 40, 200 or 400× magnification. (**B**) mRNA expression of NF-κB signaling pathway-related genes in rumen epithelium. * indicates *p* < 0.05. HC, high-concentrate group. LC, low-concentrate group.

The rumen and cecum are two microbial fermentation chambers in ruminants that can be used to study the effect of a high-concentrate diet on the gut barrier. The structure of the ruminal papilla in the LC group was relatively intact, and its structure was closely arranged. The structure of the ruminal papilla in the HC group was partially damaged, and the epithelium of the ruminal papilla in the HC group was thinner than that in the low-concentrate group at a 200× magnification (Figure 3A). Some of the stratum’s corneum was shed and the lamina propria of the ruminal papilla was less intact and embedded in a disorderly manner in the granular layer in the HC group, while the stratum corneum was more intact and the lamina propria of the ruminal papilla was deeply embedded in the granular layer, with more branches observed in the LC group under 400× magnification (Figure 3A). The mucosal layer of the ruminal wall in the HC group was thinner than that in the LC group under 40× and 200× magnification (Figure 3A). We measured the relative expression of important factors in LPS-related inflammatory response pathways in the rumen epithelium (Figure 3B). The relative expression levels of TLR-4, LBP, NF-kB, and TNF-a in the HC group were higher than those in the LC group. The relative expression of IL-1β and IL-6 of the rumen epithelium in the HC group was markedly higher than that in the LC group (Figure 3B).

**Figure 4:**
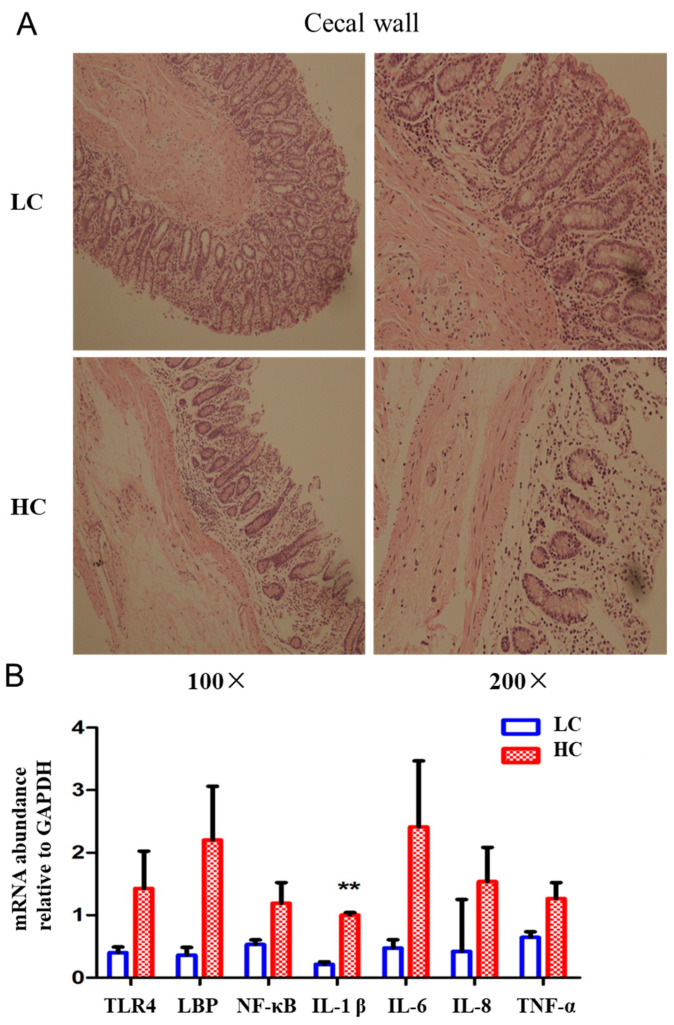
High-concentrate diet caused histopathological changes and inflammatory responses in the cecal wall. (**A**) Representative sections of the cecal wall from two groups. All sections were stained with H&E and observed at 100 or 200× magnification. (**B**) mRNA expression of NF-κB signaling pathway-related genes in cecal epthelium. ** indicates *p* < 0.01. HC, high-concentrate group. LC, low-concentrate group.

The structure of the cecum walls of cows in the LC group was relatively intact, the structure of the mucosal layer and muscular layer was relatively intact, and the large intestinal glands in the mucosal layer were arranged neatly and intactly. However, the structure of the cecal wall of cows in the HC group was damaged, the mucosal layer was thinner than that in the low-concentrate group, and the large intestinal glands were incomplete and were damaged under 100× magnification (Figure 4A). The cecum wall in the LC group had an intact mucosal layer, the mucosal epithelium could be observed, and the mucosal epithelium was composed of a single layer of columnar cells. However, the mucosal epithelium of the cecum wall in the HC group was destroyed, and the intestinal glands in the mucosal layer were incomplete under 200× magnification (Figure 4A). The mRNA level of inflammatory cytokines also showed that the expression of IL-1β in the HC group was significantly higher than in the LC group (Figure 4B). The results showed that SARA caused damage to the structure of the rumen and cecum and induced an inflammatory response in the gut barrier, which was the reason that LPS and histamine were transferred from the rumen to the bloodstream.

### 3.4. Transfer/Translocation of LPS and Histamine from the Rumen to Blood Induces an Inflammatory Response in the Liver

**Figure 5:**
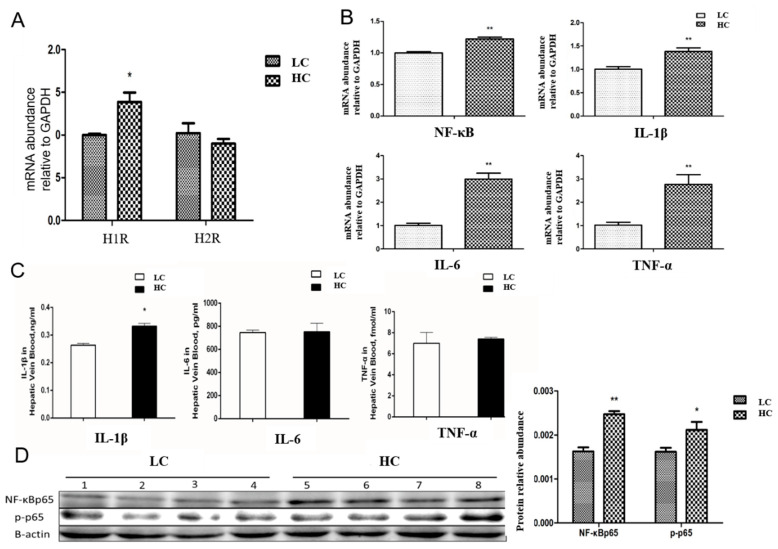
Effect of high-concentrate diet feeding on histamine receptor and inflammatory response in liver. (**A**) mRNA expression of haistamine receptor H1R and H2R in liver. (**B**) mRNA expression of NF-κB signaling pathway-related genes in liver, (**C**) the concentration of pro-inflammatory cytokines in the plasma of hepatic vein measured by Radioimmunoassay kits. (**D**) the protein expression of NF-κB p65 and phosphorylated p65 by Western Blot. * indicates *p* < 0.05 and ** indicates *p* < 0.01. HC, high-concentrate group. LC, low-concentrate group.

The physiological effects of histamine are mediated by histamine receptors, such as the H1 receptor and H2 receptor. High-concentrate diet feeding significantly increased the mRNA expression of *H1R* in the liver (Figure 5A). Another toxic metabolite, LPS, can bind to TLR4 to activate the NF-κB-regulated inflammatory signaling pathway. The level of pro-inflammatory cytokine *IL-1β* expressed in the hepatic vein was significantly increased by high-concentration feeding (Figure 5C). The mRNA expressions of *NF-κB*, *IL-1β*, *IL-6*, and *TNF-α* were markedly increased in the HC group compared with the LC group (Figure 5B). For the important inflammatory transcription factor, the protein abundance of NF-κB p65 and its activated state phosphorylated p65 were significantly increased by high-concentrate diet feeding (Figure 5D). These results showed that when histamine and LPS arrived at the liver, they induced an inflammatory response in the liver.

## 4. Discussion

The richness and diversity of ruminal bacteria declined due to the occurrence of SARA [14,28]. In this study, SARA induced by a high-concentrate diet didn’t affect the richness and diversity. Thus, the ruminal microbiota system is sensitive to diet and differs depending on the different diets. *Bacteroidetes* and *Firmicutes* are two dominant bacterial phyla in the rumen and are mainly studied [29,30]. SARA decreased *Bacteroidetes* and *Fibrobacter*, which have a high level of glycoside hydrolases (GHs) and polysaccharide lyases (PLs), and are mainly responsible for the degradation of complex polysaccharides in plants, and increased *Firmicutes* and *Proteobacteria* at phyla level [28]. Our 16S rRNA sequence result showed a similar result to one previous study that a high-concentrate diet decreased the relative abundance of *Bacteroidetes* and increased the relative abundance of *Firmicutes*. However, one review reported that *Firmicutes* are the predominant phylum whose relative abundance occupies more than 65%, and it is not affected by diet transition from high-forage to high-starch in beef cattle [31].

*Prevotella* (*Bacteroidetes* phylum), *Butyrivibrio* and *Ruminococcus* (both from *Firmicutes* phylum) are the most relatively abundant genera in the rumen [32]. SARA reduced the genus *Fibrobacter* (*Fibrobacteres* phylum) and *Ruminococcus* which belong to cellulolytic bacteria, increased *Megasphaera elsdenii* (*M. elsdenii*), which is a lactic acid utilizing bacteria, and *Prevotella* which can degrade starch and protein and produce propionate at the genus level [33]. *Lactobacillus* spp. and *Streptococcus*. *Bovis* (*S. bovis*) are the most common bacteria for starch utilization in cow’s rumen and will increase under SARA [30,31]. Another study also showed that there was an increased abundance of *M. elsdenii*, *S. bovis*, *S. ruminantium*, and *Prevotella bryantii* under a high-concentrate diet feeding in beef steers [34]. The abundance of *B. fibrisolvens* and *F. succinogenes* with fibrolytic capability was decreased in the adaption to a high-concentrate diet [31]. These studies indicate that a high-concentrate diet affects the abundance of amylolytic and fibrolytic bacteria in the rumen. Feeding a high-concentrate diet to goats increased *Butyrivibrio* levels in rumen epithelium with increased expression of Toll-like receptors [35]. However, we observed contrary results where the abundance of *Ruminococcus* increased and the abundance of *Prevotella* and *Butyrivibrio* decreased. Therefore, the changes in diverse bacteria at the phylum and genus levels are different in various kinds of studies and are also affected by the location of fluid or solid fractions, as well as whether they are loosely or tightly attached to particulates and the rumen epithelium. 

There are also some pathogenic bacteria that appear during high-concentrate diet feeding, such as *Clostridium perfringens* and *Escherichia coli* [28,36]. One study reported that *Fusobacterium necrophorum* was located in the rumen and could metabolize lactic acid and degrade epithelial protein. When cows were fed on a high-concentrate diet, the proportion of *Fusobacterium necrophorum* increased and this substance could translocate into the portal vein, resulting in liver abscesses [37]. The abundance of *Stenotrophomonas* at the genus level increased in both ruminal fluid and milk, which can cause mastitis during translocation from the rumen to the mammary gland [18]. *Proteobacteria* and *Campylobacterota* at the phylum level, as well as *Campylobacter* at the genus level, exhibited greater abundance in the ruminal epimural bacteria of the liver-abscessed cattle compared with healthy cattle. These changes and damaged ruminal walls were potentially the reason for the development of liver abscesses in cattle [38]. In future studies, the ruminal microbiota data need to be more deeply analyzed and the hepatic microbiota needs to be sequenced to explore the connection between the rumen and liver through microbiota translation.

Histamine is an important member of biogenic amines and involves many physiological processes, such as immune response, cell growth and differentiation, vessel permeability regulation, gastric juice secretion, and so on [39,40]. Some people are histamine-intolerant or -hypersensitive and a very low histamine intake can lead to severe symptoms [39]. The high level of histamine produced by the histidine metabolism of bacteria in fish causes histamine fish poisoning, which is an illness that has similar symptoms to food allergies [41]. High-concentrate diet feeding increases histamine formation, which is toxic for sheep [42]. Histamine is exclusively derived from the decarboxylation of histidine and other amino acids by histidine decarboxylase (HDC) enzymes in mammal cells [43], while this source of histamine is not significant in the rumen of bovine under normal conditions. Owing to bacterial amino acid decarboxylases, *Lactobacillus* and *Streptococcus bovis* have the ability to produce biogenic amines in dairy cows, and during high-concentrate diet feeding, the upregulation of biogenic amine in rumen may be partly due to the increased quantity of *Lactobacillus* spp. [44]. *Allisonella histaminiformans* utilize histidine decarboxylation as their sole energy source in order to produce histamine and CO_2_ as end-products [45]. In another study, *Acetitomaculum* and *Butyrivibrio* were found to be closely associated with the production of biogenic amines, including histamine, putrescine, and tyramine [46]. In this study, two important pathogenic metabolites, LPS and histamine, were measured. High-concentrate diet feeding increased the concentration of LPS and histamine in the rumen on account of changes in ruminal microbiota. Further study is needed in order to link ruminal microbiota with LPS and histamine.

Ruminal acidification to pH 5.1 induces a dramatically increased absorption of histamine in sheep fed on high-concentrate diets, and the disruption of the permeability of rumen epithelia is not due to histamine but the low pH [47]. Repeated acidosis challenges increased the concentration of LPS and histamine, while these did not increase in the plasma collected from the jugular vein in sheep [48]. SARA challenges increased biogenic amine, including histamine in ruminal fluid and peripheral blood. This effect was negatively correlated with ruminal pH, and the correlation of biogenic amine between ruminal fluid and peripheral blood was positive [44]. The morphologies of ruminal papillae and cellular junction-related genes in the rumen are compromised during high-concentrate diet feeding in dairy cattle, which means that the structural integrity of the rumen epithelium is destroyed [49]. Our previous study also reported that high-concentrate diet feeding induced the inflammation and apoptosis of rumen epithelium in dairy cows [50]. High-concentrate diet feeding can also induce increased bypass starch fermentation in the hindgut and result in a similar effect on the rumen epithelial cells [51]. In this study, we also found that the histological structures of the rumen and cecum were damaged by the high-concentrate diet and the inflammatory response happened in these parts. Therefore, LPS and histamine were transferred from the gut and arrived in the liver.

Histamine plays a role in the immunomodulation of allergy and inflammation. Histamine produced by microbiota dysbiosis in the colon suppresses NLRP6 inflammasome assembly, IL-18, and its downstream anti-microbial peptide expression, thus exacerbating dextran sodium sulfate-induced colitis [52]. Histamine treatment induced Th1 effector cell responses, increased IL-12 and IL-6 expression, and depressed MCP-1 expression in the antigen-presenting cells through the H_2_ receptor in mice [53]. The increased concentration of histamine in the blood of SARA dairy cows enhanced the adhesive ability of neutrophils, which may be the reason for the systemic inflammation in dairy cows with SARA [54]. LPS is also a major cause of systemic inflammation. Acute-phase proteins such as LPS-binding protein (LBP), serum amyloid A, and haptoglobin are usually regarded as indicators of systemic inflammation and can be diagnostic aids for SARA [54]. Even though these indicators were not measured in this study, the increased level of pro-inflammatory cytokine IL-1β in the hepatic vein also indicated the occurrence of systemic inflammation caused by the high-concentrate diet.

The important physiological effects of histamine are mediated by histamine receptors, including 4 G-protein-coupled receptors H1-H4. The H1 receptor is ubiquitously expressed and responsible for most histamine biological functions. H1 can produce allergic and asthma reactions. H2 receptor is in charge of immunomodulation, gastric acid secretion, mucus secretion, or vascular endothelial cell permeability [55]. The activation of H1R also increases vascular endothelial cell permeability, the synthesis of platelet-activating factors, and the release of nitric oxide [56]. Meanwhile, activation of H2R has more close relation with innate and adaptive immune system response through the regulation of kinds of immune cells [57,58]. The inflammatory cytokines secreted by Peyer’s patch, such as IL-4, IL-6, and IL-17, were reduced via the administration of the histamine-secreting Lactobacillus strain, and this result was reversed by H2R deficiency in mice [59]. In the trinitrobenzene sulfonic acid (TNBS)-induced mouse colitis model, the increased concentration of histamine converted into histidine by probiotic *L. reuteri* administration activated H2R signaling and suppressed acute inflammation in the colon [59]. Our previous study reported that the degree of histamine was upregulated in the ruminal fluid, lacteal artery, liver, and mammary gland by SARA occurrence. This induced the inflammatory response by H1-H4 histamine receptors in mammary gland through the H1R-Gα_q11_-PKC-NF-κB signaling pathway and the H2R-Gα_s_-PKA- NF-κB signaling pathway [26]. However, one study reported that histamine regulated the expression of H_2_R in a tissue-specific manner, while it did not affect the expression of H_1_R [60]. The diverse regulation of histamine in the immune system may depend on the complex system of immunoregulation, determined by the expression and activity receptor subtypes. We found that histamine increased the expression of H1R and that LPS activated the NF-κB signaling pathway, inducing the inflammatory response in the liver.

## 5. Conclusions

This study showed that feeding with a high-concentrate diet for 18 weeks induced SARA and changed the microbiota’s composition and community. The gut structure was damaged, and the inflammatory response happened in the rumen and cecum. Under SARA and a microbiota imbalance, LPS and histamine were transferred into bloodstream through the gut barrier, and this induced systemic inflammation. These two toxic metabolites also arrived in the liver and activated the NF-κB signaling pathway. Thus, our study offered a basis for the view that the ruminal microbiome disorder has an effect on the health of dairy cows and we can manipulate the health of dairy cows by regulating the ruminal microbiota in the future. The limitations of this study were that the connection between the change of ruminal microbiota and LPS/histamine needed to be analyzed deeply and the hepatic microbiota needed to be sequenced in order to explore the connection between the rumen and liver through microbiota translation. The experimental method limited the distinction between the cellular source and microbial source of histamine.

## Figures and Tables

**Table 1:** Ingredients, nutrient composition, and forage-to-concentrate ratio (F:C) of the diets used in this experiment.

Ingredient	Diets	
	LC	HC
Corn silage	30	20
Alfalfa hay	30	20
Corn	24.3	32
Bran	0	12.4
Soybean meal	13.5	13
Calcium phosphate dibasic	0.85	0.45
limestone	0	0.8
Salt	0.35	0.35
Premix ^a^	1	1
F:C	6:4	4:6
Nutrient composition		
NEI, MJ/kg CP ^b^	6.36 16.99	6.71 16.92
EE ^b^	3.93	4.07
NDF ^b^	36.54	31.45
ADF ^b^ NFC ^b^	22.51 33.76	17.56 39.32
Ca ^b^	0.88	0.89
P ^b^	0.43	0.43

^a^ Premix contained 5.25 g/kg of Fe, 1.2 g/kg of Cu, 5.5 g/kg of Mn, 13 g/kg of Zn, 50 mg/kg of Co, 27 mg/kg of Se, 170 mg/kg of I, 1900 ku/kg of vitamin A, 250 ku/kg of vitamin D, and 3 g/kg of vitamin E. HC, high-concentrate group. LC, low-concentrate group. ^b^ %DM.

**Table 2:** Ruminal pH in rumen and LPS concentrations in rumen, cecum contents, plasma of the portal and hepatic veins.

Item	LC	HC	SEM	*p*-Value
Daily average pH	6.02	5.90	0.03	0.03
Duration of pH < 5.6 per day, min/d	99.0	223	30.3	<0.01
Mean LPS in ruminal contents (EU/mL)	4.921 × 10^5^	7.855 × 10^5^	0.644 × 10^5^	<0.05
Mean LPS in cecum contents (EU/g)	11.960 × 10^5^	13.115 × 10^5^	0.149 × 10^5^	<0.01
Mean LPS in plasma of portal vein (EU/mL)	0.106	0.204	0.019	<0.01
Mean LPS in plasma of hepatic vein (EU/mL)	0.067	0.053	0.004	>0.05

HC, high-concentrate group. LC, low-concentrate group.

**Table 3:** Content of histamine in the ruminal liquid, jugular vein and liver of the LC group and the HC group in dairy cows.

Histamine Content (ng/mL)	Groups		SEM	*p*-Value
	LC	HC		
Ruminal liquid	1386.94	2575.38	259.74	<0.01
Plasma of Jugular vein	1644.75	2380.50	149.08	<0.01
Liver	133.22	183.03	4.57	<0.01

HC, high-concentrate group. LC, low-concentrate group.

**Table 4:** Effects of high-concentrate (HC) diet feeding on the average richness and diversity of ruminal bacterial community (*n* = 6).

Items	LC	HC	SEM	*p*-Value
OTUs	852.83	847.83	7.51	0.756
ACE	876.72	890.41	6.01	0.275
Chao1	887.03	908.70	5.87	0.060
Shannon index	5.65	5.45	0.06	0.100
Simpson index	0.01	0.01	0.001	0.207

OUTs, operational taxonomic units. ACE, Chao1, Shannon index and Simpson index are diversity indexes. HC, high-concentrate group. LC, low-concentrate group.

**Table 5:** Bacteria at the genus level with significant differences between two groups.

Phylum	Family	Genus	LC	HC	SEM	*p*-Value
Bacteroidetes	Prevotellaceae	Hallella	0.26	0.10	0.03	0.001
		Prevotella	22.69	15.98	2.02	0.041
		Unclassified_Prevotellaceae	1.99	1.42	0.13	0.010
	Unclassified_Bacteroidetes	Unclassified_Bacteroidetes	2.02	5.21	1.19	0.027
Firmicutes	Lachnospiraceae	Unclassified_Lachnospiraceae	0.45	1.19	0.08	<0.001
	Ruminococcaceae	Oscillibacter	1.02	1.75	0.14	0.007
		Ruminococcus	0.74	1.26	0.11	0.015
		Sporobacter	1.10	0.73	0.08	0.012
	Unclassified_Clostridia	Unclassified_Clostridia	0.26	0.39	0.04	0.037
	Acidaminococcaceae	Succiniclasticum	0.75	0.29	0.08	0.006
Planctomycetes	Planctomycetaceae	Unclassified_Planctomycetaceae	0.11	0.21	0.02	0.022
Spirochaetes	Spirochaetaceae	Unclassified_Spirochaetaceae	0.19	0.31	0.01	<0.001

HC, high-concentrate group. LC, low-concentrate group.

## Data Availability

Data are contained within the article.
